# Supplementary figures and images for: MITO-VATION: Feasibility of a technology-supported structured home exercise program in Mitochondrial Disease
Source: PLOS Digit Health. 2026 Feb 26;5(2):e0001257. doi: 10.1371/journal.pdig.0001257 (PMC12944747; doi:10.1371/journal.pdig.0001257)

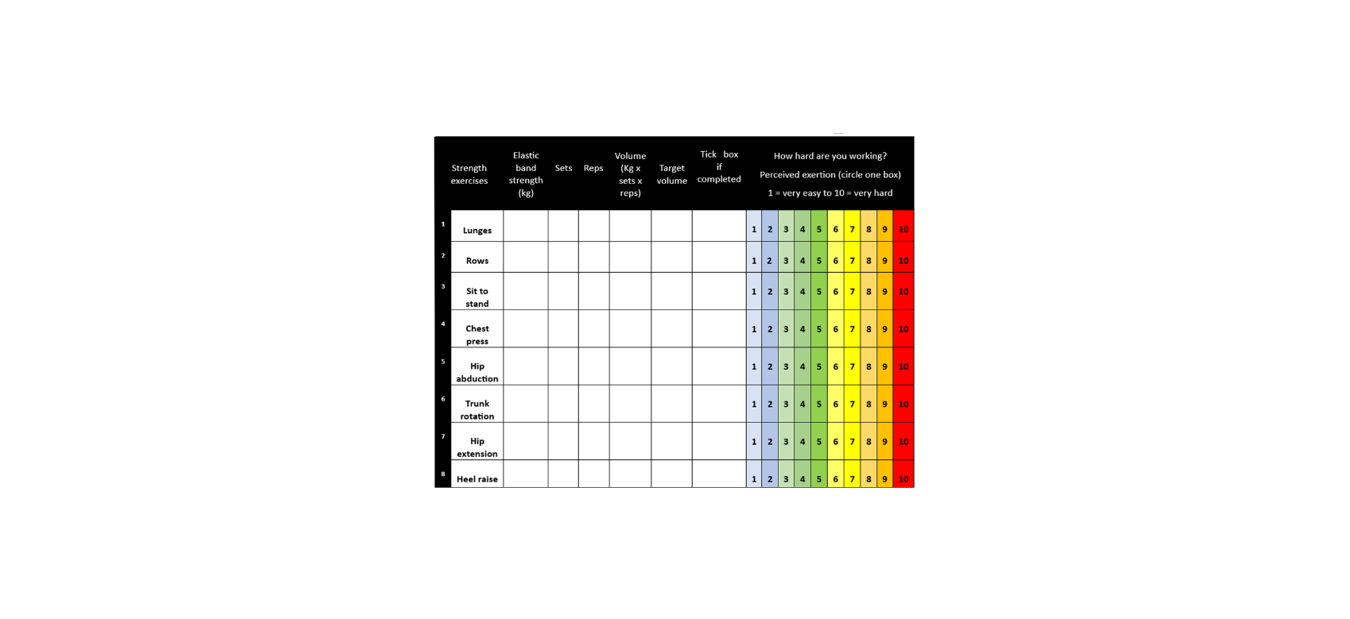

Supplement: S1 Fig — (TIFF) [file pdig.0001257.s001.tiff]

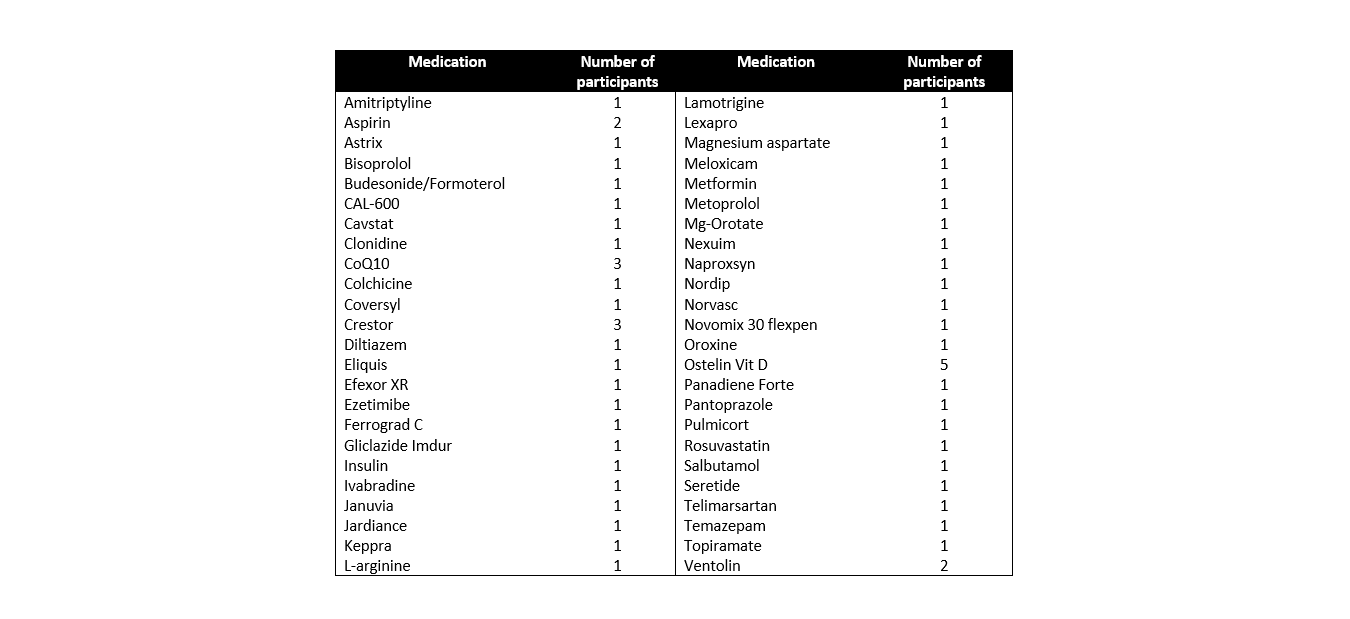

Supplement: S1 Table — (TIFF) [file pdig.0001257.s002.tiff]

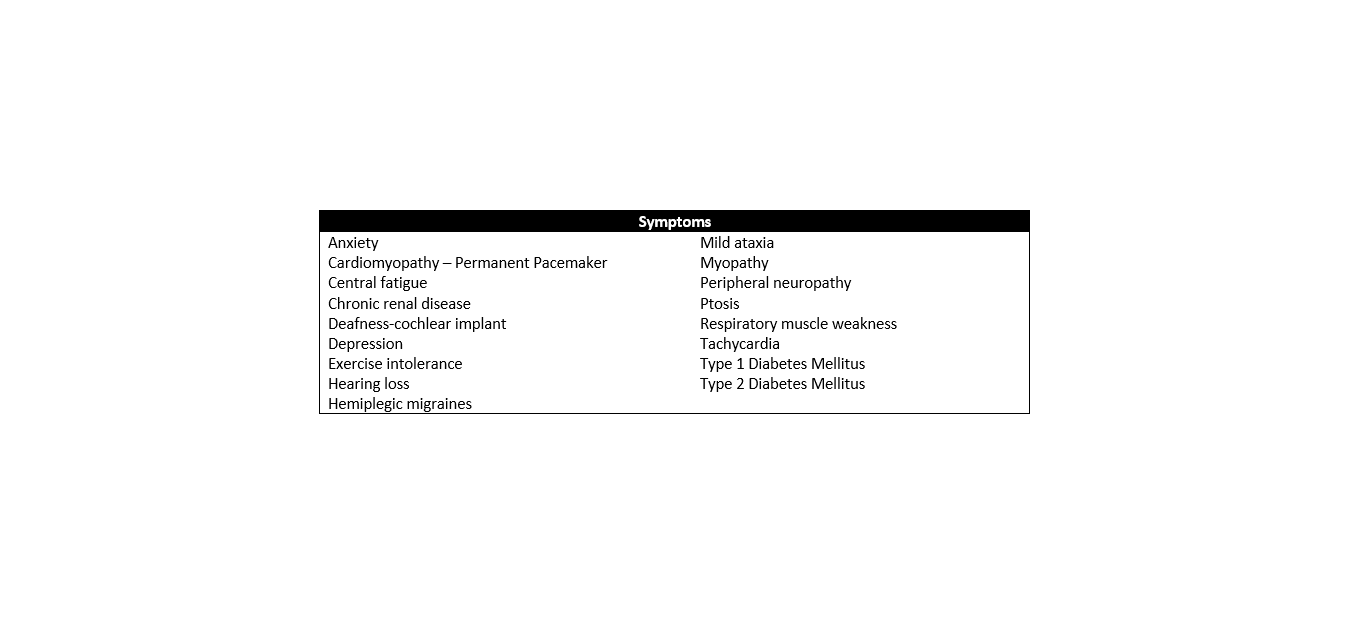

Supplement: S2 Table — (TIFF) [file pdig.0001257.s003.tiff]

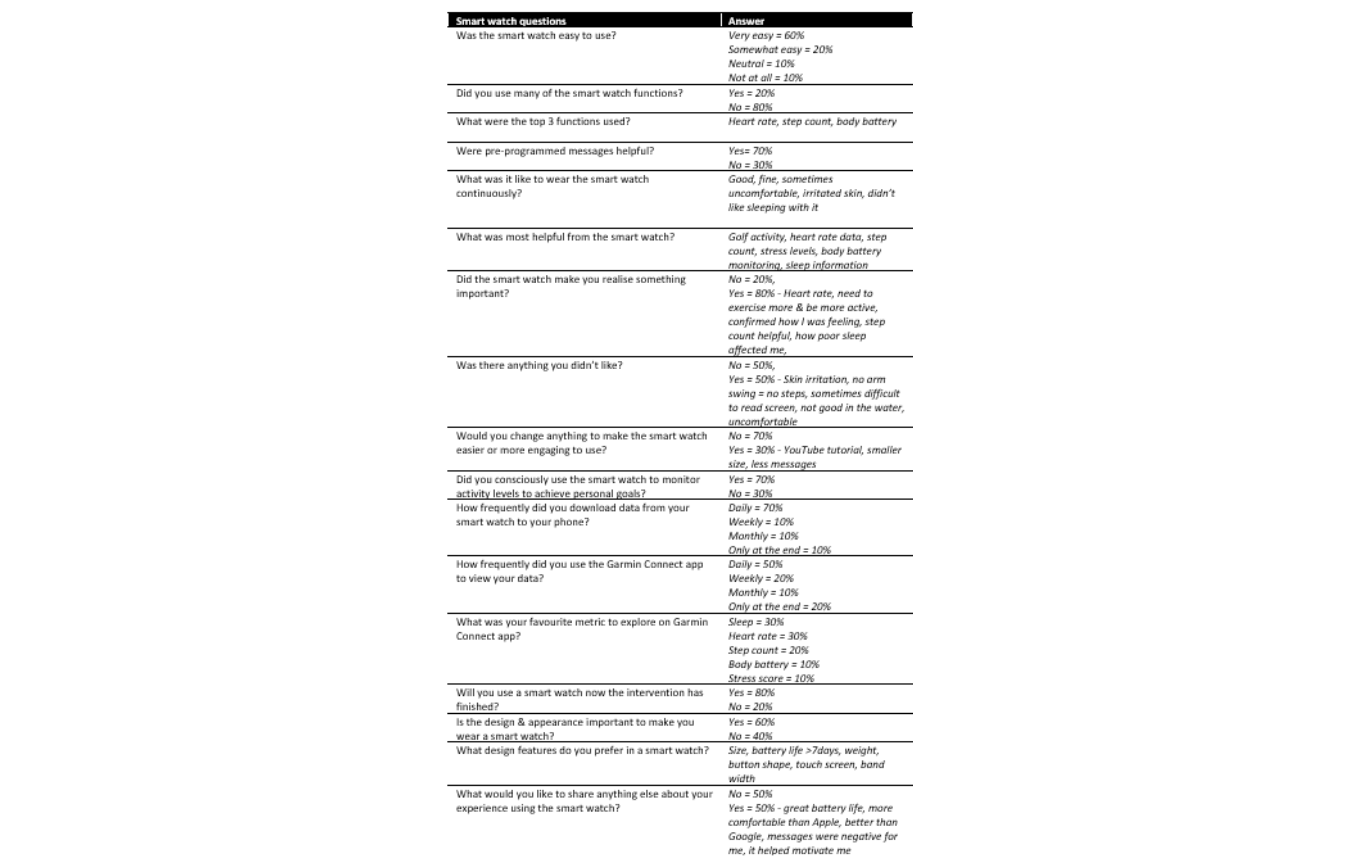

Supplement: S3 Table — (TIFF) [file pdig.0001257.s004.tiff]

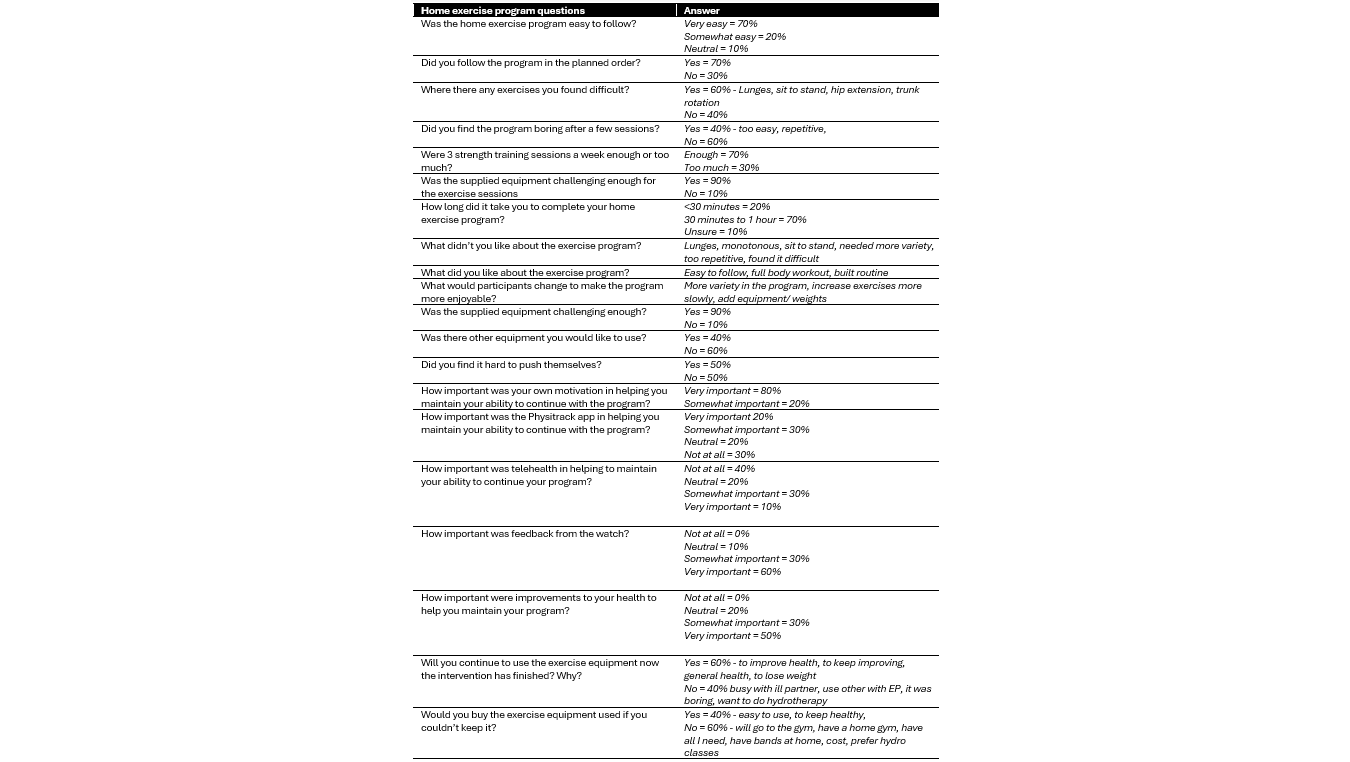

Supplement: S4 Table — (TIFF) [file pdig.0001257.s005.tiff]

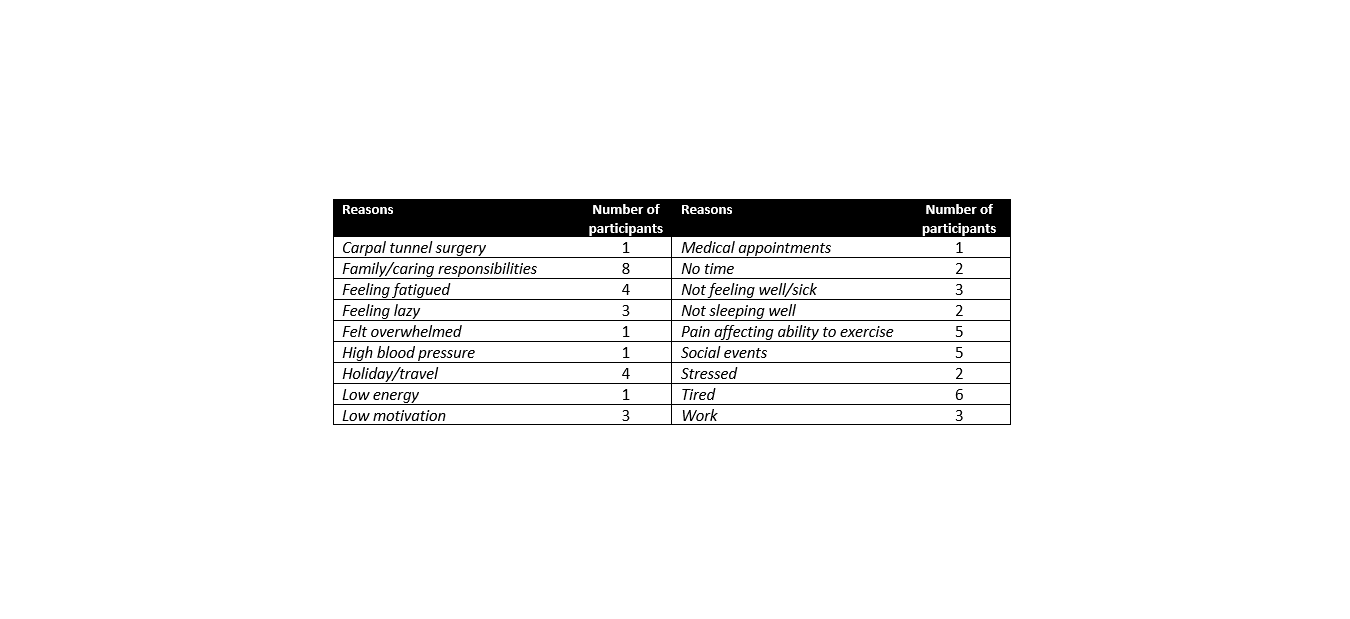

Supplement: S5 Table — (TIFF) [file pdig.0001257.s006.tiff]

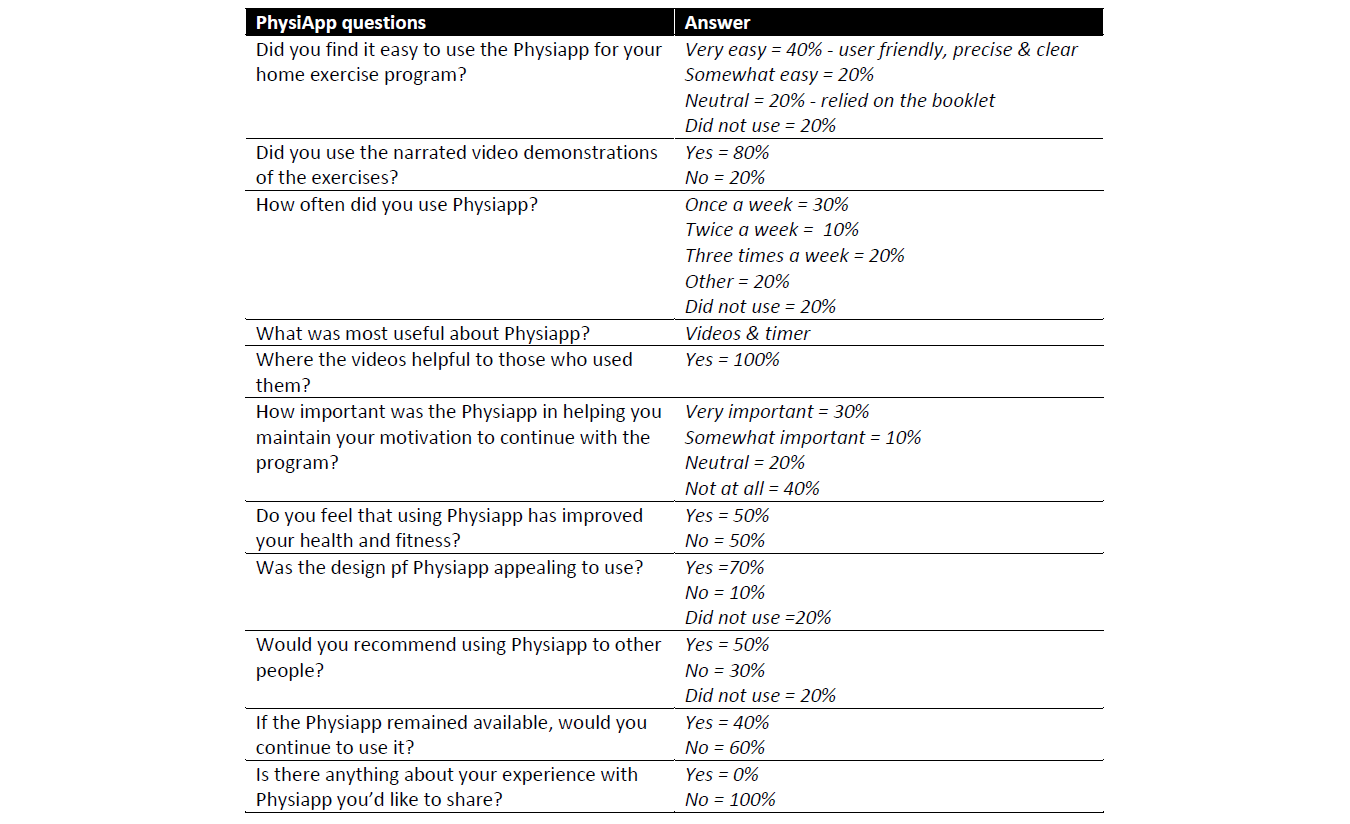

Supplement: S6 Table — (TIFF) [file pdig.0001257.s007.tiff]
