# Supplementary material for: MITO-VATION: Feasibility of a technology-supported structured home exercise program in Mitochondrial Disease
Source: PLOS Digit Health. 2026 Feb 26;5(2):e0001257. doi: 10.1371/journal.pdig.0001257 (PMC12944747; doi:10.1371/journal.pdig.0001257)
Supplement: Attachment — Submitted filename: Response to Editor and Reviewers 14.12.25.docx [file pdig.0001257.s008.docx]

**Response to Editor and Reviewers**

**Manuscript Title:** **MITO-VATION -- Feasibility of a technology-supported structured home exercise program in Mitochondrial Disease (PDIG-D-25-00320)**

**Authors:** Jeremey T Horne, Natalie E Allen, Serene S Paul, Judith S Walker, Carolyn M Sue

**Date:** 14/12/25

Dear Editor,

We thank you and the reviewers for the thoughtful and constructive feedback on our manuscript. We have carefully addressed all comments and believe the manuscript is substantially improved as a result. Below, we provide a point-by-point response to each comment and indicate the changes made to the manuscript.

In addressing the editor and reviewer comments, we also took the opportunity to make this work more concise and improve flow. As such, there are some sections highlighted as changes that were only due to editorial changes rather than to content changes in response to the reviewer and editor comments.

Thank you for your consideration of our revised manuscript.

Sincerely,

Jeremey T Horne, BAppSc (Physio) On behalf of all authors

**RESPONSE TO EDITORIAL REQUIREMENTS**

**1.** Amend detailed Financial Disclosure statement with author initials and funder role.

**Response:** We have revised the Financial Disclosure statement (lines 554-556, p23) as follows:

*"JH received a research fellowship grant from the Mito Foundation. NA, SP, JW and CS received no funding for this study. The funders had no role in study design, data collection and analysis, decision to publish, or preparation of the manuscript."*

**2.** Complete Competing Interests statement.

**Response:** We have added a Competing Interests statement (line 559, p24):

*"The authors have declared that no competing interests exist."*

**3.** Ensure Ethics Statement is available at the beginning of Methods section with required elements.

**Response:** We have created a new "Ethics Statement" subsection at the beginning of the Methods section (lines 135-137, p3):

*“This study was approved by the South Eastern Sydney Local Health District Human Research Ethics Committee (HREC 2024/ETH00250). Written consent was obtained from all participants prior to data collection.”*

**4.** Clarify data sharing for first-party data.

**Response:** We have provided a Data Availability statement (line 559, p24):

*“Study data is available in supplement 9.”*

**5.** Remove all trademark/copyright symbols (®, ©, TM). Provide written permission for identifiable person in Supplements 1 and 2 or remove photographs.

**Response:** We have removed from the manuscript all instances of trademark symbols including Garmin®, PhysiTrack®, PhysiApp®, and Garmin Connect®.

Permission for the identifiable person in Supplement 1 has been granted by Physitrack. They have provided a confirmation letter which we have retained on record. We have provided a footnote to Supplement 1 stating this permission (line 838, p36).

Please note that we have consolidated Supplements 1 and 2 into Supplement 1 in the revised manuscript.

**RESPONSE TO REVIEWER #1**

We thank Reviewer #1 for the suggestions to improve the clinical detail of our manuscript.

**Comment 1:** "In baseline assessment of participants, please mention which medication did they receive; for example, did they receive any AED, corticosteroids, vitamins, Coenzyme Q10, L-carnitine, anti-arrhythmic drugs?"

**Response:** Participants did not receive any specific medications as part of this study; they continued taking their usual medications (lines 186-187, p5). We have now added a complete list of all participants' medications as Supplement 3 (lines 840, p36) and have referenced Supplement 3 in the baseline characteristics section of the results (lines 311, p10).

**Comment 2:** "Were there any modifications about the dietary regiment of participants during the trial period? Or did they ever receive?"

**Response:** No dietary modifications were made during the trial. This information has been added to the manuscript in the intervention section of the methods (line 186-187, p5).

**Comment 3:** "Please mention the gender of participants."

**Response:** Gender information is available in Table 1 (lines 317-317, p10-11) and is also mentioned in text (line 313, p10).

**Comment 4:** "Since this study measures the grip of participant, it’s important to mention which participants are right or left-handed and compare the results of non-dominant hand of each participant with their dominant one."

**Response:** Thank you for this suggestion. We have updated Table 3 (lines 418-419, p17) to indicate grip strength outcomes according to hand dominance.

**RESPONSE TO REVIEWER #2**

We thank Reviewer #2 for the insightful comments that have helped us strengthen the interpretation and presentation of our feasibility data.

**Comment 1:** "I'm not totally clear how to interpret the feasibility 'goals', most of which were not met, yet the study concludes the intervention 'may be feasible'. How low of adherence would mean the intervention is not feasible?"

**Response:** Our conclusion that the intervention "may be feasible" is based on a combination of four feasibility measures, not just adherence alone. We assessed feasibility across four distinct domains of recruitment, retention, adverse events and adherence to intervention targets, as described below.

1. Recruitment Rate: The target for feasibility was 50%, but our study achieved 26%. This was largely attributable to geographical barriers, as most eligible participants lived outside metropolitan Sydney and could not attend 2 in-person appointments within 9 weeks. In real-world clinical practice with flexible scheduling, recruitment would likely improve. This indicates a need for system-level changes rather than intervention non-feasibility.

2. Retention Rate: The target was 80% and our study achieved 100%. Zero dropouts demonstrate excellent retention. It shows that once enrolled, participants found the DHT acceptable to use.

3. Intervention-Related Adverse Events: No serious adverse events, and only four minor adverse events, related to the intervention were reported: 1 participant reported muscle soreness, 3 reported flare-ups of pre-existing arthritis. All were successfully managed and did not result in dropouts, as participants continued with slight modifications to their program, demonstrating safety of the program and feasibility of remote monitoring.

4. Adherence to Intervention Targets: This feasibility measure showed mixed results. The target for the 5 sub-components of adherence was set at 80%, as shown in table 2 (lines 334-336, p13). However, adherence was only achieved in 2 of the 5 sub-components: 80% of participants met the watch wear and weekly catch-up sessions targets. Participants failed to achieve adherence for daily step, weekly intensity minutes or strength exercise session targets.

Overall, when considering all feasibility measures together, 50% were fully met, with valuable learnings from those not met. The high retention rate, excellent safety profile, and adherence to the passive intervention components (watch wear, telehealth catch-ups) suggest the DHT approach is feasible. The lower adherence to active exercise components indicates a need for program modifications rather than fundamentally indicating non-feasibility of the intervention.

Importantly, our post hoc analysis revealed that participants with pre-existing exercise habits achieved 4/5 intervention goals, while non-exercisers achieved 0/5 goals. This suggests the intervention is feasible for individuals with exercise experience and requires substantial modification for sedentary individuals with PMD. We have added this exploratory post hoc analysis in the methods (lines 298-299, p10) and results (lines 376-378, p15; and Table 2, lines 334-336, p13).

In summary, we have modified the methods to clarify the combined feasibility outcomes (lines 258-268, p8) and reworded the results (lines 323-336, p12-13) and discussion (lines 422-429, p18) to provide a clearer interpretation of the intervention’s feasibility.

**Comment 2:** "Compliance drops from 87% to 23% for strength sessions over time - any indication is this because of exercise intolerance, failure of engagement in the app, something else?"

**Response:** This is a critical observation, and data from our post-intervention questionnaires explain this decline. Primary reasons from participant feedback included 40% found the program monotonous and mundane after a few weeks while 30% reported that 3 sessions per week was too much for them. There was a need for more variety in exercise selection. Secondary contributing factors from exercise diaries included fatigue (condition-related), lack of motivation (particularly on low-energy days), illness, pain flares (from pre-existing arthritis), social events, family obligations and carer duties, work commitments, medical appointments and poor sleep. Our data shows that 3 weeks represents a critical time point where exercise programs need modification to prevent adherence decline.

We have modified the results (lines 393-410, p16) and provided detail in Supplement 7 to include reasons for the drop in exercise compliance. We have also modified the discussion (lines 475-483, p20) to describe both reasons for the lack of exercise compliance and clinical implications to mitigate such issues, including highlighting the need for more frequent program adjustments every 2-3 weeks.

**Comment 3:** "I am unclear what 'monitoring' actually means in this study - e.g., how was the monitoring (line 246-248) incorporated into the weekly catch-up calls or other contacts, in terms of reviewing actual data with participants?"

**Response:** Monitoring in this case referred to the data being uploaded daily by participants and being reviewed by the physiotherapist via their Garmin Connect account at any time. This typically occurred 2 to 3 times throughout the week to make sure participants were downloading their data so it could be fully assessed at the end of each week to set the following week’s targets and establish talking points for the catch-up session. If data was not uploaded the participants would receive an email reminder. We have clarified monitoring in the Methods (lines 242-251, p7-8).

**Comment 4:** "I would actually bring the self-report survey data into the main paper, it might actually provide the most learning opportunity for readers in this space interested in building better experiences."

**Response**: We agree that the acceptability data provides crucial insights for researchers and clinicians developing similar interventions. We have updated the "Acceptability of the intervention" section in the Results that summarises key findings from the questionnaires. However, we recognize this could be enhanced. We have made the following improvements, expanded the "Acceptability of the intervention" section of the results (lines 381-412, p16-17) to provide more details and included representative participant quotes to illustrate key themes.

**Minor Comment A:** "I am curious why it makes sense to benchmark PMD against general exercise recommendations... Is there any specific data addressing that exercise levels for 'all adults' applies to PMD patients?"

**Response:** We acknowledge that current PMD guidance emphasises individualised, symptom- and test-guided prescriptions, particularly for moderate to severe disease. Our study population had mild to moderate PMD without substantial disability. For this group, we believed that benchmarking against Australian general population exercise recommendations was appropriate. We used population-level recommendations as a framework but still individualised the intervention based on baseline functional capacity, medical history and comorbidities, and weekly monitoring data, participant symptoms and feedback.

**Minor Comment B:** "The figures and tables were extremely grainy making them hard to read."

**Response:** We apologise for the poor image quality in the submitted version. All figures and tables have been updated to high resolution (minimum 300 dpi).

**Minor Comment C:** "Was it surprising that 2 of the 10 seem to have 3-4 hours of intense activity per day (Figure 3)?"

**Response:** This observation highlights the complexity of using heart rate-based intensity metrics in populations with comorbidities. These seemingly high levels were not due to actual intense exercise, but rather a physiological phenomenon related to diabetes. The two participants with unusually high weekly intensity minutes (Figure 3) had diabetes. Diabetes is associated with autonomic nervous system dysfunction, particularly elevated resting heart rate (often >80 bpm in our participants), which lowered the thresholds for entering "intensity zones" and allowed accumulation of intensity minutes during routine, non-exercise activity. We have updated the Results (lines 350-352, p14) and Discussion (lines 466-468, p19) sections of the paper to explain this.

**Minor Comment D:** "Sounds like the participants could choose video or phone, but the term 'Telehealth' refers just to video for some reason? Line 347. Can the authors provide a sentence as to why video was not acceptable?"

**Response:** We have now clarified that "Telehealth" includes video consultation via PhysiApp platform, traditional telephone calls and emails. Participants could choose their preferred method. We have updated the ‘4) Weekly catch-up session target’ section of the results (lines 243-251, p7-8) to detail what constituted telehealth.

**Minor Comment E:** "Not sure I would use drop-out rate as a measure of acceptability. e.g., if compliance were zero but the subjects simply did not take the step to actually drop out, this does not seem to argue the protocol was acceptable?"

**Response:** We have clarified in the Methods that retention rate was one of four components of the feasibility measure (primary outcome) (lines 259-268, p8). We distinguish acceptability measures (from post-assessment questionnaires that probed acceptability of specific intervention components) from the feasibility outcome (that combined recruitment, retention, adverse events and adherence to intervention targets). We have updated the results to reflect this distinction (feasibility outcomes reported in lines 323-378, p12-15; acceptability reported in lines 381-412, p16-17).

**RESPONSE TO REVIEWER #3**

We thank Reviewer #3 for the helpful suggestions to strengthen the manuscript's context and methodology.

**Comment 1:** "To better establish the context and significance of the research, please consider adding recent statistics on the worldwide prevalence and impact of PMD."

**Response:** Thank you for this suggestion. We have added statistics related to global prevalence as well as the significant public health impact in Australia to justify the need for scalable, accessible interventions like DHT-supported exercise program. We have modified the Introduction as follows (lines 86-90, p1):

*“The global prevalence of PMD is estimated to be approximately 1 in 5,000 individuals, although rates vary between populations due to diagnostic and genetic differences [3]. In Australia, it is estimated that at least 1 in every 200 people carry a disease-causing mitochondrial DNA mutation [4]. The estimated average annual cost of PMD was AU $112,721 per household [5].”*

**Comment 2:** "Lines 121-122: Please provide a clear rationale for selecting the Garmin Vivoactive 5 smartwatch and the PhysiTrack platform. It would be helpful to mention if these specific tools have been validated or used in previous research and to briefly explain why they were considered optimal for this study's objectives compared to other available options."

**Response:** We have added validation evidence and described the practical benefits of these tools to the methods (Garmin: lines 163-167, p4; and Physitrack: lines 177-181, p5).

**Comment 3:** "Lines 124, 177, 179: There appears to be a discrepancy regarding the study duration. The text states an 8-week period, but the mention of 'Week 0' for baseline and 'Week 9' for follow-up suggests a total duration of 10 weeks. Please clarify the exact timeline of the intervention and data collection to ensure consistency."

**Response:** Thank you for raising this discrepancy. We have updated the manuscript to clarify that the study (i.e. intervention) was conducted over 8 weeks, described that baseline and follow-up assessments were conducted immediately prior to or following the 8-week intervention period, and removed mention of all numbered weeks.

**Comment 4:** "The authors should clarify if there were any significant differences in baseline measures among the participants. If so, please discuss how these differences were controlled for in the analysis or how they might have influenced the study's outcomes."

**Response:** We have carefully considered this suggestion. However, as this was a small study of 10 participants, formal statistical analysis of how differences at baseline would over- interpret results.

However, there were notable differences in participants’ exercise history (30% regular exercisers, 70% non-exercisers). We therefore conducted an exploratory post hoc analysis which showed that participants who had exercised in the past appeared to have better adherence to the intervention. We have updated the Methods (lines 298-299, p10), Results (lines 375-377, p15) and Discussion (lines 480-483, p20) to include this post hoc analysis.

Please note also that Table 1 has been replaced with the table that was formerly supplement 7, to provide aggregated descriptive data of participant baseline characteristics (lines 316-317, p11-12).

**Comment 5:** "The manuscript would be strengthened by an analysis of the potential influence of age on the study's outcomes. I would recommend conducting a supplementary statistical analysis to explore this. For instance, the authors could stratify participants into two or three age-based categories and compare the outcomes using an appropriate test. Alternatively, grouping participants by other relevant variables—such as NMDAS score, exercise history, or clinical presentation—and examining the role of age within these groups could also yield valuable information."

**Response:** As per our response to Comment 4, there is insufficient data to conduct robust statistical analyses to explore the influence of age on the study outcomes. We have, however, noted in the Limitations section (lines 523-535, page 22) the need for larger studies that can explore if age influences exercise performance in this population.
